# The Effect of a 13-Valent Conjugate Pneumococcal Vaccine on Circulating Antibodies Against Oxidized LDL and Phosphorylcholine in Man, A Randomized Placebo-Controlled Clinical Trial

**DOI:** 10.3390/biology9110345

**Published:** 2020-10-22

**Authors:** Hendrika W. Grievink, Pim Gal, Maria Ozsvar Kozma, Erica S. Klaassen, Johan Kuiper, Jacobus Burggraaf, Christoph J. Binder, Matthijs Moerland

**Affiliations:** 1 Centre for Human Drug Research, Zernikedreef 8, 2333 CL Leiden, The Netherlands; wgrievink@chdr.nl (H.W.G.); pgal@chdr.nl (P.G.); eklaassen@chdr.nl (E.S.K.); kb@chdr.nl (J.B.); 2 Division of BioTherapeutics, Leiden Academic Center for Drug Research, Leiden University, Einsteinweg 55, 2333 CC Leiden, The Netherlands; j.kuiper@lacdr.leidenuniv.nl; 3 Clincal Pharmacy and Toxicology, Leiden University Medical Center, Albinusdreef 2, 2333 ZA Leiden, The Netherlands; 4 Department of Laboratory Medicine, Medical University of Vienna, 1090 Vienna, Austria; maria.ozsvarkozma@meduniwien.ac.at (M.O.K.); christoph.binder@meduniwien.ac.at (C.J.B.); 5 Department of Surgery, Leiden University Medical Center, Albinusdreef 2, 2333 ZA Leiden, The Netherlands; 6 Division of Pharmacology, Leiden Academic Center for Drug Research, Leiden Universtiy, Einsteinweg 55, 2333 CC Leiden, The Netherlands

**Keywords:** cardiovascular disease, vaccine, clinical trials, translational medicine, atherosclerosis, oxLDL, phosphorylcholine

## Abstract

**Simple Summary:**

Atherosclerosis is the main underlying mechanism for cardiovascular disease. The main cause for atherosclerosis development is oxidized low density lipoprotein (oxLDL) accumulation in the vessel wall and a subsequent immune response. It has been established that immunoglobulin M antibodies against oxLDL help protect against atherosclerosis. It has been found in mice that vaccination with *Streptococcus pneumoniae* results in an increase of these protective antibodies and thereby decreases the development of atherosclerosis. In this study, we investigated if this increase of antibodies can be found in human as well. Twenty-four healthy male volunteers were vaccinated with Prevenar-13, a pneumococcal vaccine, using different dosing regimens. An increase in anti-Prevenar antibodies was found, showing that the vaccination worked. However, no increase in protective anti-phosphorylcholine or anti-oxLDL antibodies was observed. This work shows that vaccination against pneumococcal does not seem to be a suitable treatment option to help prevent atherosclerosis development, although further research would be required to test alternative pneumococcal-based vaccines, vaccination regimens or study populations.

**Abstract:**

In mice vaccination with *Streptococcus pneumoniae* results in an increase in anti-oxLDL IgM antibodies due to mimicry of anti-phosphorylcholine (present in the cell wall of *S. pneumoniae*) and anti-oxLDL IgM. In this study we investigated the human translation of this molecular mimicry by vaccination against *S. pneumoniae* using the Prevenar-13 vaccine. Twenty-four healthy male volunteers were vaccinated with Prevenar-13, either three times, twice or once in a double-blind, placebo-controlled, randomized single center clinical study. Anti-pneumococcal wall, oxLDL and phosphorycholine antibody levels were measured at a fixed serum dilution, as well as circulating lipid levels over the course of 68 weeks. A significant increase in anti-oxLDL IgG and IgM was seen in the group receiving two doses six months apart compared to the placebo. However, these differences were not observed in the groups receiving a single dose, two doses one month apart, or three doses. This study shows that vaccination with Prevenar-13 does not result in robust anti-oxLDL IgM levels in humans. Further research would be required to test alternative pneumococcal-based vaccines, vaccination regimens or study populations, such as cardiovascular disease patients.

## 1. Introduction

Oxidized low density lipoprotein (oxLDL) particles play a key role in the etiology of atherosclerosis [1]. In the vessel wall, oxLDL is recognized and phagocytosed by macrophages primarily via scavenger receptors leading to foam cell formation [2]. Macrophage foam cells are hallmark cells of atherosclerotic lesions and participate in the inflammatory responses that mediate smooth muscle cell migration and proliferation, and extracellular matrix production, and thereby stimulate atherosclerotic plaque progression. 

Several mouse studies showed that IgM antibodies against oxLDL are atheroprotective [3,4]. IgM antibodies against oxidized particles facilitate the clearance of apoptotic cells, thereby promoting the resolution of inflammation [5,6]. Additionally, these antibodies neutralize the proinflammatory effects of oxidized phospholipids [7,8]. Inhibition of scavenger receptor-mediated oxLDL uptake by macrophages prevents the formation of foam cells and subsequent progression of atherosclerotic plaque formation [3,9]. In clinical studies, oxLDL-specific IgM has been reported to be a protective factor for atherosclerosis development, correlating with cardiovascular disease incidence and clinical outcome [10,11,12,13].

In contrast to IgM, the role of oxLDL–specific IgG in atherosclerosis is thought to be atherogenic. OxLDL–IgG complexes have been shown to induce survival of plaque-resident monocytes [14] and secretion of proatherogenic cytokines by mast cells [15]. Clinical studies showed a correlation between oxLDL–IgG antibodies and acute coronary syndrome, suggesting an untoward role of this antibody in plaque destabilization [16]. In human, oxLDL–specific IgG antibody titers correlated inversely to the oxLDL serum concentration [17] and, in mouse, serum cholesterol levels [18], which suggests that oxLDL–specific IgG facilitates phagocytosis of oxLDL by macrophages.

Mouse experiments showed that certain IgM clones binding oxLDL bind phosphorylcholine (PC) of oxidized—but not unoxidized—phospholipids [3,19]. Importantly, Binder et al. showed in Ldlr knockout mice that vaccination against *S. pneumoniae* using pneumoccocal extracts induced high titers of oxLDL–specific IgM, subsequently leading to a decrease in atherosclerotic lesions [19]. This effect was explained by the fact that PC is present as part of the capsular polysaccharide of *S. pneumoniae*. Moreover, immunization with PC conjugated to carrier proteins also induced oxLDL–IgM and decreased the extent of atherosclerosis in ApoE knockout mice [20,21].

Autoantibodies against PC are also found in humans, where low levels of PC–IgM autoantibodies correlate with a higher incidence of cardiovascular disease [22,23,24,25]. Moreover, pneumococcal-specific IgG and oxLDL-specific antibody titers correlated significantly in subjects who had received pneumococcal vaccination [26], although there are also reports of an absent effect of pneumococcal vaccination on oxLDL–specific IgM levels [27]. 

The present proof-of-concept study investigated the human translation of the observed effects of pneumococcal immunization in mice. Healthy human volunteers were vaccinated with a 13-valent conjugated pneumococcal vaccine (Prevenar-13^®^), and the induction of PC- and oxLDL-specific antibodies was measured.

## 2. Materials and Methods

This investigation was a double-blind, randomized, placebo-controlled, parallel, single-center study with twenty-four healthy males between 18 and 45 years of age. The study was performed at the Centre for Human Drug Research in Leiden, The Netherlands. Participants were recruited via advertisements and social media. Participants were assessed to be generally healthy based on a complete medical screening and had no previous exposure to the 13-valent pneumococcal vaccine. All participants gave written informed consent prior to any study-related activity. The study was approved by the Ethics Committee of the Leiden University Medical Centre (LUMC) and Declaration of Helsinki principles were followed. The study is registered in the Dutch Trial Registry (Nederlands Trial Register, NTR) under study number NTR5643 and took place for all participants simultaneously between March 2016 and October 2017. This study was funded by the European Union, call FP7-HEALTH-2013-INNOVATION, project ID 603131.

### 2.1. Vaccination Schedule

The 13-valent conjugated pneumococcal vaccine (Prevenar-13^®^) used in this study was from a single batch (batch no. MU7958). The presence of residual PC in the vaccine preparation was confirmed by ELISA using the PC-specific mAb IgM E06. Placebo consisted of 0.9% NaCl solution. Since there are clear visual differences between these vaccinations, three physicians were unblinded for administration of the vaccine. These physicians were not otherwise involved in the study. 

**Figure 1:**
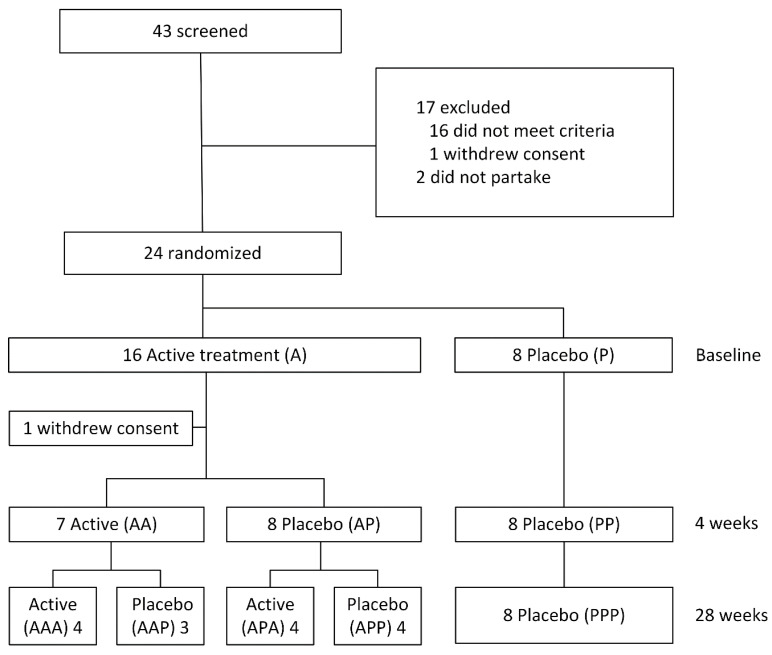
Study flowchart.

Vaccinations took place at three time points: at baseline, at four weeks and at 28 weeks. Subjects were randomized in a consecutive order based on eligibility. The randomization code was generated using SAS v9.4 for Windows (SAS Institute Inc., Cary, NC, USA) by an independent statistician. The randomization code was only made available for data analysis after study completion. There were five different treatment arms, as displayed in Figure 1. In the mouse study three immunizations were enough for oxLDL–specific IgM induction [19]. In the mouse study proteinase-treated *S. pneumoniae* extracts were used. In this design, the power to detect differences between placebo and active treatment arms was optimized between baseline and at the 28 week time point (PP vs. AA vs. AP, *n* = 8 per group).

### 2.2. Antibody Measurements

K2EDTA plasma antibody levels to Prevenar, PC–BSA, and CuSO4-oxidized LDL (oxLDL) were measured by chemiluminescent ELISA as reported previously [28]. In brief, Prevenar (Pfizer) was coated at 1:5000, PC–BSA (Biosearch Technologies, Novato, CA, USA) and oxLDL at 5 ug/ml in PBS/EDTA. IgM antibodies were measured at a dilution of 1:500 and IgG antbodies at 1:1000. Binding of IgG subclasses to Prevenar was measured at a dilution of 1:100 and to PC–BSA at 1:100 for IgG3 and IgG4, and 1:500 for IgG2.

Serum levels of total cholesterol, low density lipoprotein (LDL), high density lipoprotein (HDL) and triglycerides were measured by the chemistry lab of the Leiden University Medical Center on the Cobas P800 analyzer (Hoffmann–La Roche, Basel, Switzerland).

### 2.3. Power Calculation

In humans, the median anti-oxLDL IgG levels in the healthy, unvaccinated population is around 50 U/l, with an interquartile range of around 25–75 U/l [26]. Anticipating an immune response minimally resulting in a five-fold rise in IgG and IgM antibody levels, and based on an inter-subject variability of 50% in basal IgG and IgM levels [19,24,26], a sample size of 4–8 subjects per group (dependent on the contrast) will be sufficient to meet the study objectives. This was a conservative approach considering in the magnitude of the oxLDL-specific IgM response observed in the murine model [19].

### 2.4. Statistical Analysis

Data are presented as mean ± standard deviation (SD). In case of non-normal distribution, parameters were log-transformed. Repeatedly measured variables were analyzed with a mixed model analysis of variance with fixed factors treatment group, time and the interaction of treatment group and time as fixed factor and subject as random factor. Primary endpoints (Prevenar-specific Ig levels, oxLDL-specific Ig levels and PC-specific Ig levels) were compared between treatment groups for the 0–4 week window, the 4–28 week window, and the 28–68 week window. As a secondary endpoint, lipid levels in circulation were measured. Estimated differences were calculated between the groups. A positive value indicates a higher estimated value for the active group, a negative value indicates a lower value for the active group. The analysis was performed in SAS v9.4 (SAS Institute, Cary, NC, USA).

## 3. Results

Twenty-four healthy volunteers were included in the study; their baseline characteristics can be found in Table 1. One subject withdrew consent after two weeks for non-study related reasons (Figure 1). This subject was randomized to the active-active-placebo treatment arm and was not replaced.

### 3.1. Anti-Prevenar Antibodies

**Figure 2:**
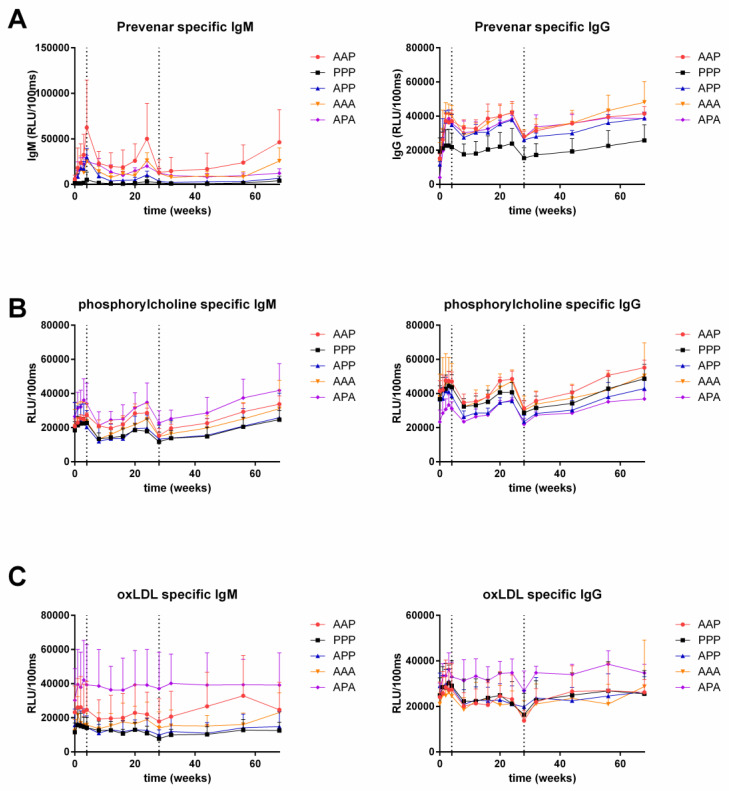
Anti-Prevenar (quantified by antibodies against pneumococcal wall saccharide) (**A**); anti-phosphorylcholine (**B**); and anti–oxLDL responses (**C**). Mean + SD; *n* = 4 per group, *n* = 3 for AAP (active-active-placebo) group and *n* = 8 for PPP (placebo-placebo-placebo) group. RLU/100ms: relative light units/100 ms ‘A’: active treatment, ‘P’: placebo treatment. Dotted lines indicate vaccination times (baseline, 4 weeks, 28 weeks). Statistical analysis using a mixed model analysis of variance with fixed factors: treatment group and time, and the interaction of treatment group and subject as random factor. For *p*-values for Prevenar responses, see Table 2. No significant changes were found for oxLDL and PC responses, except for PC-specific IgM in the APA (active-placebo-active) group compared to PPP (*p* = 0.005), and oxLDL-specific IgM and IgG responses at 68 weeks in the APA group compared to PPP (*p* = 0.007 for IgG, *p* = 0.005 for IgM).

Prevenar-specific IgG was significantly increased in all subjects who received any active treatment compared to placebo-treated subjects, see Table 2 and Figure 2A. Prevenar-specific IgM was significantly increased in subjects who received any active treatment up to 28 weeks, however after 68 weeks only subjects receiving three active doses had a significantly increased IgM level compared to placebo.

### 3.2. Anti-OxLDL and Anti-PC Antibodies

No difference was observed in PC-specific IgG levels compared to placebo, for any of the active treatment groups. Similarly, no difference was observed for PC-specific IgM levels between active treatment groups and placebo, with the exception of IgM levels being higher in the APA group compared to PPP during the study period (ED: 9409.7, 95% CI: 3227.5–15,591.9, *p* = 0.005) (Figure 2B). 

There were no differences in oxLDL-specific IgG and IgM antibodies between active and placebo treated subjects up to 28 weeks. However, at 68 weeks, subjects who received an active treatment at baseline and after 28 weeks (APA) had an increased oxLDL-specific IgG level compared to subjects receiving three placebo injections (PPP) with an estimated difference (ED) of 9913 (95% CI: 3141–16,686; *p* = 0.007). As shown in Figure 2C difference between these groups were also observed for oxLDL-specific IgM levels (ED: 12235, 95% CI: 4179–20,290; *p* = 0.005).

### 3.3. Lipids

**Figure 3:**
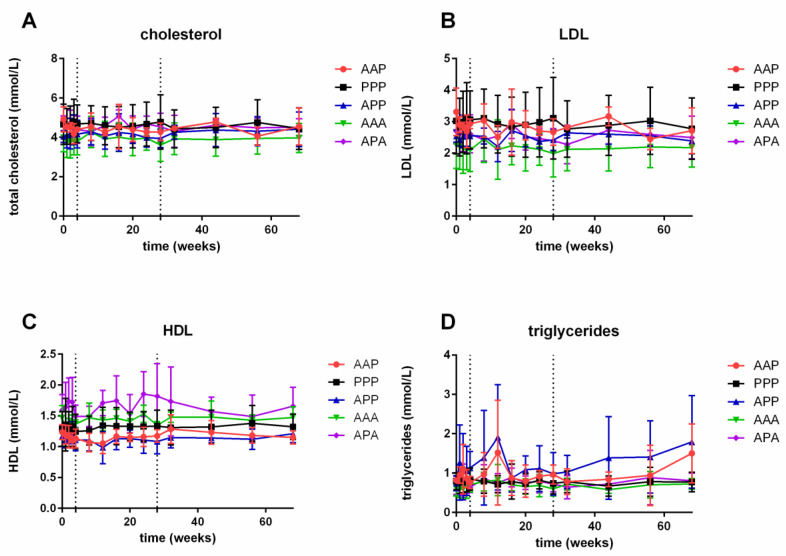
Cholesterol (**A**), LDL (low density lipoproteins) (**B**), HDL (high density lipoproteins) (**C**) and triglycerides plasma levels (**D**) (mean + SD). *n* = 4 per group, *n* = 3 for AAP group and *n* = 8 for PPP group. ‘A’: active treatment, ‘P’: placebo treatment. Dotted lines indicate vaccinations (baseline, 4 weeks, 28 weeks). Statistical analysis using a mixed model analysis of variance with fixed factors: treatment group and time, and the interaction of treatment group and subject as random factor. No significant differences were found, with the exception of APP versus PPP for triglycerides (*p* = 0.009).

The levels of total cholesterol (**A**), LDL (**B**), HDL (**C**) and triglycerides (**D**) of all groups during the study are depicted in Figure 3. No significant differences were found between treatment groups, with the exception of subjects receiving a single active treatment at the beginning of the study (APP) who had a significant higher triglyceride level compared to placebo (PPP) (ED: 7.9%, 95% CI 18%–171%; *p* = 0.009).

## 4. Discussion

The present study evaluated the effect of a 13-valent conjugate pneumococcal vaccine on the induction of anti-oxLDL and anti-PC antibodies and cholesterol levels in humans. Several vaccination regimens were tested, where subjects received either one, two or three doses of Prevenar-13 over a period of 28 weeks compared to placebo. Despite the induction of an adequate anti-Prevenar 13 antibody response, there was no evident induction of either PC-specific or oxLDL-specific antibodies. Prevenar-13 immunization induced a significant IgG2 response when subjects were immunized at least twice, while levels of IgG3 and IgG4 were not altered (Figure S1). In one active treatment group a statistically significant difference in PC-specific and oxLDL-specific antibody levels was observed compared to placebo. This occurred in the group receiving two doses of the vaccine at the start of the study and after 28 weeks (APA). A significant increase in PC-specific IgM and oxLDL-specific IgM and IgG was observed at 68 weeks. Interestingly, we observed a significant increase in PC-specific IgG3 for the group receiving two vaccinations four weeks apart (AAP) compared to placebo (PPP) (Figure S1 panel B). Elevated oxLDL-specific IgM is believed to be atheroprotective [10,11,12,13], but the role of oxLDL-specific IgG levels is not fully elucidated. Laczik et al. [16] showed that increased oxLDL-specific IgG levels correlate with acute coronary syndrome, while immunization with oxLDL, resulting in an increase in oxLDL-specific IgG, resulted in decreased plaque development in several mouse models [18]. Furthermore, oxLDL levels are inversely correlated with oxLDL-specific IgG serum levels [17]. Binder et al. [19] showed *S. pneumoniae* immunization in mice induced a much stronger oxLDL-specific IgM response than an oxLDL-specific IgG response (100,000 vs. 10,000 RLU/100ms). The current clinical data are at odds with this observation, though the observed anti-oxLDL responses in the APA group may indicate that the timing of vaccination could be important. 

Previous studies have investigated the 23-valent polysaccharide pneumococcal vaccine as a means to elicit oxLDL-specific antibodies in humans, with conflicting results [26,27,29]. One study reported that, after vaccination with the 23-valent vaccine, an increased oxLDL-specific IgG antibody titer was observed compared to healthy, unvaccinated individuals (248 U/l vs. 55 U/l). An effect of vaccination on IgM was not reported. In the other two studies, no association between vaccination status and oxLDL-specific antibodies was observed. There are, however, key differences between these studies and the present study. First, patients in all three referenced studies only got a single vaccination, whereas in the present study, up to three vaccinations were given. Second, there is a major difference between the 23-valent polysaccharide vaccine that was used in these studies and the 13-valent polysaccharide conjugate vaccine that was used in the present study. The latter is constituted of cell wall polysaccharides that have been conjugated to a protein. The 13-valent vaccine is thereby considered to be more powerful in eliciting an antibody response against *Streptococcus* pneumonia [30,31]. These data were the basis for the selection of the 13-valent vaccine for the current clinical study. 

Although the sample size per group was relatively small in the current study (*n* = 4 for treatment groups, *n* = 8 for placebo), the study was sufficiently powered to detect Prevenar-13-induced rises in IgM titers, had these occurred as in the murine experiments [19].

The induction of anti-pneumococcal wall saccharide antibodies demonstrates that the 13-valent vaccine was effective for its intended use. However, the vaccine did not elicit a robust oxLDL-specific IgM response, as observed in mouse experiments. One explanation for the poor induction of oxLDL-specific antibody responses by Prevenar 13 may be that the murine immune response is poorly translatable to humans. Human and mice have numerous discrepancies in their innate and adaptive immune systems, such as cytokine receptor and costimulatory molecule expression and function [32,33]. Moreover, murine studies are commonly performed in inbred strains, with limited genetic variability between mice. This, and the fact that mice are kept in a more-sterile environment, results in a smaller immune diversity compared to humans [34]. On the other hand, the mild but significant induction of oxLDL-specific antibody responses in one active treatment group (APA) does support further clinical investigation of mimicry between pneumococcal vaccination and oxLDL. Furthermore, it could be hypothesized that the PC content in the Prevenar-13 vaccine used was not high enough to induce robust PC- and oxLDL-specific responses.

## 5. Conclusions

Vaccination of humans with Prevenar-13 did not significantly increase PC-specific antibodies and oxLDL-specific antibodies nor resulted in significant changes in plasma lipids. Nevertheless, subgroup analyses suggested an induction of PC-specific and oxLDL-specific IgM and IgG in individuals receiving two doses six months apart. Future research should investigate alternative pneumococcal vaccines (driving more significant anti-PC antibody responses), vaccination regimens, or study populations, to confirm or refute the hypothesis that molecular mimicry underlying pneumococcal-driven anti-oxLDL responses as observed in mice occurs in humans.

## Figures and Tables

**Table 1:** Baseline characteristics.

Parameter	*n* = 24
Age (years)	28.5 ± 8.5
Gender male (%)	100
Ethnicity Caucasian (%)	100
Height (cm)	180.5 ± 5.3
Weight (kg)	75.0 ± 11.0
BMI (kg/m^2^)	23.0 ± 3.2
Heart rate (min^−1^)	58.5 ± 9.0
Systolic blood pressure (mmHg)	123 ± 9.3
Diastolic blood pressure (mmHg)	75.1 ± 6.7

**Table 2:** Estimated differences for Prevenar-specific IgG and IgM levels.

	IgG			IgM		
	ED	%95 CI	*p* Value	ED	%95 CI	*p* Value
0–4 weeks (A vs. P)	19,187.8	14,098.7–24,276.9	<0.0001	13,128.5	5356.9–20,900.1	0.0020
4–28 weeks (AP vs. PP)	16,656.9	11,626.7–21,687.1	<0.0001	5480.2	1143.1–9817.3	0.0158
4–28 weeks (AA vs. PP)	17,155.5	12,449.3–21,861.7	<0.0001	9272.0	4568.5–13,975.5	0.0005
28–68 weeks (AAA vs. PPP)	22,733.6	17,512.7–27,954.5	<0.0001	8189.1	1407.0–14,971.1	0.0209
28–68 weeks (AAP vs. PPP)	18,439.7	12,869.1–24,010.2	<0.0001	10,320.1	−23.1–20,663.3	0.0505
28–68 weeks (APA vs. PPP)	25,805.3	19,200.6–32,409.9	<0.0001	987.0	−6717.0–8691.0	0.7903
28–68 weeks (APP vs. PPP)	17,116.7	11,720.9–22,512.5	<0.0001	2233.9	−4392.3–8860.1	0.4862

ED = estimated difference, A = active, P = placebo, CI = confidence interval.

## Supplementary Materials

The following are available online at https://www.mdpi.com/2079-7737/9/11/345/s1, Figure S1: Prevenar-specific and phosphorylcholine-specific IgG subclass responses.
