# Supplementary material for: The Effect of a 13-Valent Conjugate Pneumococcal Vaccine on Circulating Antibodies Against Oxidized LDL and Phosphorylcholine in Man, A Randomized Placebo-Controlled Clinical Trial
Source: Biology (Basel). 2020 Oct 22;9(11):345. doi: 10.3390/biology9110345 (PMC7716233; doi:10.3390/biology9110345)
Supplement: Supplementary file 1 [file biology-09-00345-s001.zip › supplemental figure (1).pdf]

Supplemental figure

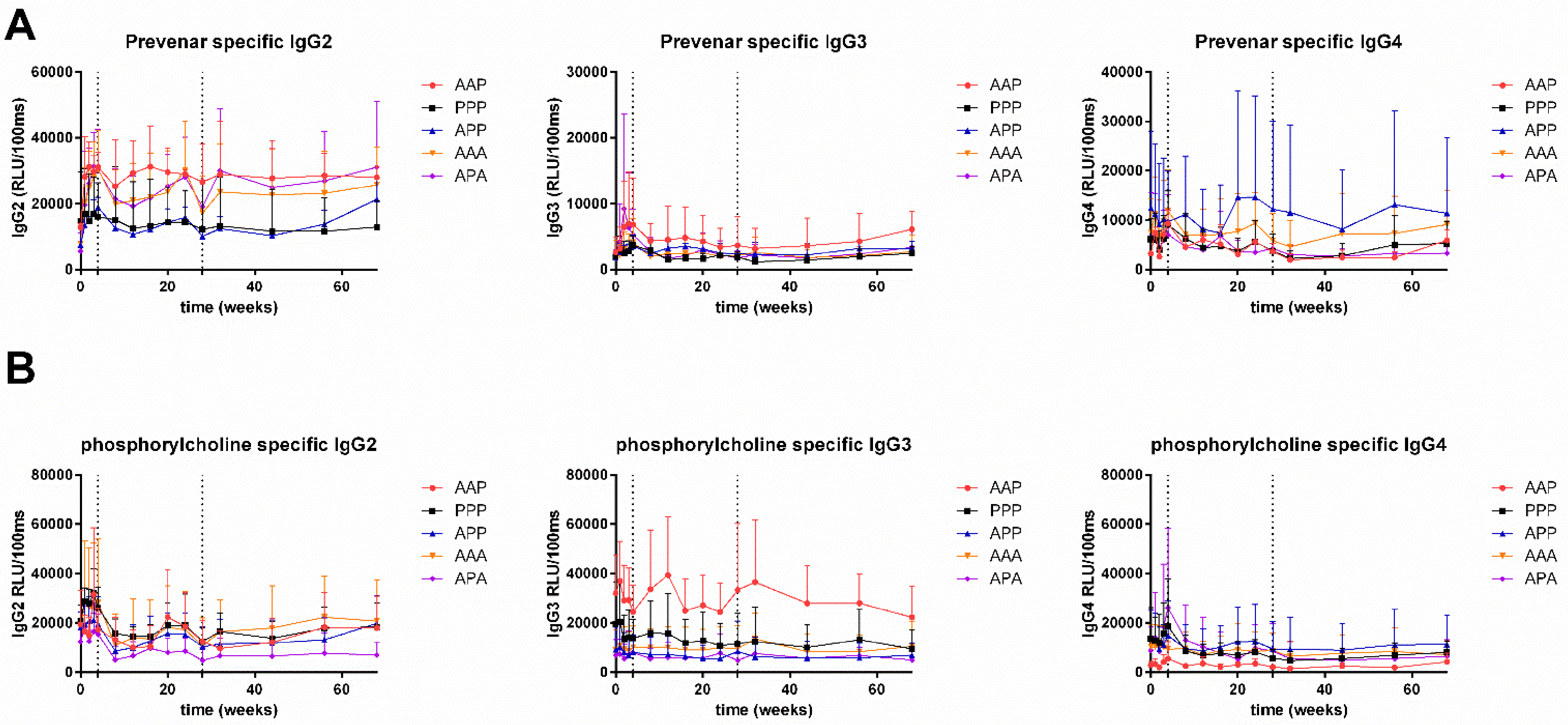

Figure S1: Prevenar-specific (panel A) and phosphorylcholine-specific (panel B) IgG subclass responses (mean + SD). N=4 per group, n=3 for AAP, n=8 for placebo. RLU/100ms: relative light units/100ms. 'A': active treatment, 'P': placebo treatment. Dotted lines indicate vaccinations (baseline, 4 weeks, 28 weeks). Statistical analysis using a mixed model analysis of variance with fixed factors: treatment group and time, and the interaction of treatment group and subject as random factor.
